# Circulating cardio-enriched microRNAs are associated with long-term prognosis following myocardial infarction

**DOI:** 10.1186/1471-2261-13-12

**Published:** 2013-02-28

**Authors:** Olof Gidlöf, J Gustav Smith, Kazuma Miyazu, Patrik Gilje, Anna Spencer, Sten Blomquist, David Erlinge

**Affiliations:** 1 Department of Cardiology, Faculty of Medicine, Lund University, Skåne University Hospital, SE-221 00, Box 117, Lund, Sweden; 2 Department of Clinical Sciences, Faculty of Medicine, Lund University, Malmö, Sweden; 3 Broad Institute of Harvard and MIT, Cambridge, MA, USA; 4 Department of Cardiothoracic Surgery, Anaesthesia and Intensive Care, Faculty of Medicine, Lund University, Lund, Sweden

**Keywords:** MiRNA, Myocardial infarction, Acute coronary syndrome, Biomarker, Prognosis

## Abstract

**Background:**

Increased levels of cardio-enriched microRNAs (miRNAs) have been described in patients with myocardial infarction (MI). We wanted to evaluate the diagnostic and prognostic potential of cardio-enriched miRNAs in patients presenting with a suspected acute coronary syndrome (ACS).

**Methods:**

Cardio-enriched miRNAs (miR-1, miR-208b and miR-499-5p) were measured using real time PCR in plasma samples from 424 patients with suspected ACS treated in a coronary care unit. miRNAs were assessed for discrimination of a clinical diagnosis of myocardial infarction and for association with 30-day mortality and diagnosis of heart failure. Correlation with left ventricular systolic dysfunction as measured by the ejection fraction (LVEF) was also assessed. To confirm myocardial origin miRNA was measured during coronary artery bypass surgery.

**Results:**

miRNAs were higher in MI patients and correlated with LVEF (p < 0.001). Discrimination of MI was accurate for miR-208b (AUC = 0.82) and miR-499-5p (AUC = 0.79) but considerable lower than for Troponin T (AUC = 0.95). Increased miRNA levels were strongly associated with increased risk of mortality or heart failure within 30 days for miR-208b (OR 1.79, 95% CI = 1.38-2.23, p = 1 × 10^-5^) and miR-499-5p (OR 1.70, 95% CI = 1.31-2.20, p = 5 × 10^-5^) but the association was lost when adjusting for Troponin T. During surgery miR-208b and miR-499-5p was released in the coronary sinus after cardioplegia-reperfusion to markedly higher levels than in a peripheral vein.

**Conclusions:**

Our findings confirm increased levels of cardio-enriched miRNAs in the blood of MI patients and establish association of increased miRNA levels with reduced systolic function after MI and risk of death or heart failure.

## Background

MicroRNA (miRNA) is a class of short non-coding RNA, which function as moderators of gene expression [1-3]. miRNA binds to complementary sequences in the 3’ untranslated region of mRNA and thereby facilitates down regulation of gene expression. The expression of miRNA is in itself subject to tight regulation, both temporally and spatially [4], and some miRNA species are highly tissue specific [5-7]. Cardio-enriched miRNAs play a crucial role in cardiac development [8,9] and have been associated with the development of dilated cardiomyopathy [10]. In the event of tissue damage or stress, it is believed that cells release some of their miRNA content into the circulation [11-14]. The fact that most miRNA species are remarkably stable and readily detectable in blood makes them excellent candidate biomarkers for various diseases, including myocardial infarction and heart failure [15-18].

Biomarkers play a central role in the diagnosis and prognostication in myocardial infarction, but current biomarkers like creatine kinase MB (CKMB) and cardiac troponins have several shortcomings, including slow release patterns [19] or limitations in specificity [20]. The prospect of using circulating cardio-enriched miRNA as biomarkers for myocardial infarction has been explored in several studies [21-24]. However, most studies conducted thus far have been small and focused on the acute phase of the disease. We assessed the usefulness of three cardio-enriched circulating miRNAs, miR-1, -208b and -499-5p, previously shown to be elevated in plasma following myocardial infarction, both for distinguishing myocardial infarction (MI) from non-MI chest pain in the acute phase and for long-term prognosis of death and development of heart failure in a large population of patients presenting with acute coronary syndrome (ACS). Moreover, to confirm the myocardial origin of these miRNAs, we analysed the miRNA content in the coronary sinus before and immediately after cardioplegia in patients undergoing coronary artery bypass surgery.

## Methods

### CABG patient samples

Four patients undergoing coronary artery bypass grafting (CABG) were included in the study. Patient characteristics are described in Table 1. Blood samples were taken from the coronary sinus before and immediately after cardioplegia. As a control, blood was also sampled from a peripheral artery immediately after cardioplegia. Plasma was prepared by centrifugation at 1500*g for 15 minutes and stored at -80°C until analysis.

**Table 1:** CABG Patient characteristics

**Characteristic**	**Patient 1**	**Patient 2**	**Patient 3**	**Patient 4**
**Age**	61	84	65	80
**Sex**	M	M	F	M
**Diabetes Mellitus**	Y	N	N	N
**Hypertonia**	Y	N	Y	Y
**Smoking**	C	E	E	E
**Preoperative MI**	Y	N	N	Y
**Aortic Valve Stenosis**	N	Y	Y	N

*M* = Male, *F* = Female, *Y* = Yes, *N* = No, *C* = Current smoker, *E* = Ex-smoker.

### ACS patient samples

All patients presenting with suspected ACS pain in the coronary care unit at Skane University Hospital in Lund were offered to take part in the study. See Table 2 for patient characteristics. ST-elevation myocardial infarction (STEMI) diagnosis was based on ECG criteria, non-STEMI (NSTEMI) on troponin levels together with clinical symptoms of ischemia. Non-MI was defined as absence of troponin-elevation and absence of ST-elevation. A peripheral venous blood sample was drawn into tubes containing EDTA, plasma was prepared as described above and stored in liquid nitrogen until analysis. 71% of blood samples were taken within 24 hours, 82% within 48 hours and 93% within 72 hours. In 90% of the patients, samples were obtained after interventional therapy. 95% of patients were treated with percutaneous coronary intervention (PCI). Left ventricular ejection fraction (LVEF) was assessed with echocardiography before discharge. Troponin T was measured using a standard clinical high sensitivy troponin method. Samples were taken at admission, at 3, 10 and 20 hours. Daily repeated samples were taken until a peak value had been identified.

**Table 2:** ACS Patient characteristics

**Characteristic**	**Distribution total cohort**	**Distribution experienced endpoint**	**Distribution no end-point**	**p**
**Sample size**	407	74	333	
**Age**	65 (11.1)	67.8 (11.8)	64.5 (10.9)	**0.02**
**Men**	76.9%	78.1%	76.6%	0.79
**Diabetes Mellitus**	17.2%	17.8%	17.1%	0.88
**Hypertonia**	43.5%	52.1%	41.6%	0.10
**Current Smoking**	24.8%	23.3%	25.1%	0.74
**Mean time to sample** (hours)	38.4 (32.2)	42.7 (13.7)	38.2 (6.9)	0.28
**Diagnosis**				**0.04**
STEMI	42.5%	53.4%	40.1%	0.79
NSTEMI	35.9%	35.6%	35.9%	
Non-MI	21.6%	11.0%	24.0%	
Unstable angina	63,6%	85.7%	62,5%	
Stable angina	21,6%	0%	26.2%	
Chest pain	14,8%	14.3%	11.2%	
**Troponin T** (ng/l)	2.55 (3.76)	5.14 (5.25)	1.98 (3.08)	**<0.0000001**
**Concurrent therapy**				
Aspirin	35.1%	34.2%	35.3%	0.79
Beta-adrenergic antagonists	32.4%	56.2%	29.9%	**0.05**
Ca^2+^-channel antagonists	17.7%	19.2%	17.4%	0.61
Statins	31.7%	35.6%	30.8%	0.60
ACE inhibitors	17.7%	26.0%	15.9%	0.08
**Death within 3 months** (%)	1.2%	6.8%	0%	**0.000001**
**LVEF**				**<0.0000001**
>50%	62.8%	5.6%	78.7%	
40-49%	17.4%	5.6%	21.3%	
30-39%	14.5%	67.6%	0%	
<30%	4.4%	21.1%	0%	
**miRNA** (ln 2^-∆Ct^)				
miR-1	−2.69 (0.99)	−2.58 (1.08)	−2.71 (0.97)	0.32
miR-208b	−4.59 (2.22)	−3.70 (2.38)	−4.78 (2.14)	**0.0001**
miR-499-5p	−4.77 (1.80)	−4.03 (2.04)	−4.93 (1.71)	**0.00009**

Continuous variables are presented as sample mean and standard deviation. P-values reflect comparisons between “Experienced endpoint” and “No endpoint” and are derived from Student’s t-tests for continuous variables and from Pearson’s chi-squared tests for frequency distributions. P-values below the threshold for statistical significance are bolded. *STEMI*, ST-elevation myocardial infarction; *NSTEMI*, non ST-elevation myocardial infarction; *MI*, myocardial infarction; *LVEF*, left ventricular ejection fraction.

### Sample preparation and laboratory analysis

Before analysis, plasma samples were thawed on ice and 250 μl aliquots were mixed 1:3 with TRIzol LS (Invitrogen, Carlsbad, USA). Samples were vortexed for >30 seconds and total RNA (including miRNA) was isolated using the miRNeasy kit (Qiagen, Hilden, Germany) according to the manufacturer’s instructions. cDNA was prepared using miRCURY LNA Universal cDNA synthesis kit (Exiqon, Vedbaek, Denmark) according to the manufacturer’s instructions. Genomic DNA contamination was assessed with no-reverse transcriptase controls. Quantitative real-time polymerase chain reaction (qRT-PCR) was carried out in 10 μl duplicate reactions, using Fast SYBR Green Master Mix (Applied Biosystems, Carlsbad, USA) with LNA primer sets (Exiqon) specific for the three human cardio-enriched miRNAs miR-1 (gene symbol: MIR1, miRBase accession number: MIMAT0000416) miR-208b (MIR208A, MIMAT0004960) and miR-499-5p (MIR499A, MIMAT0002870) as well as the control miRNA miR-17 (MIR17, MI0000071) and 4 μl of 80× diluted cDNA preparation on a StepOnePlus Real-Time PCR System (Applied Biosystems). Thermal cycling consisted of an initial denaturation at 95°C for 20 s, followed by 40 cycles of 95°C for 3 s and 60°C for 30 s. A melt curve was performed after each PCR. All PCR reactions yielded a single peak on the melt curve, indicating acceptable specificity of the primers. No template controls were added on each plate and in case of amplification in these wells the plate was rerun. Calibration curves were run to validate each assay. For miR-1 slope = -3.42, y-intercept = 40.23, r^2^ = 0.99, efficiency = 96%, linear range = 6.55*10^5^ - 40 copies, limit of detection (LOD) = 40 copies, Ct standard deviation at LOD = 0.15, intraassay variation = 0.03, for miR-208b, slope = -3.38, y-intercept = 38.57, r^2^ = 0.98, efficiency = 97.7%, linear range = 2.6*10^6^-10 copies, LOD =10 copies, Ct standard deviation at LOD = 0.59, intraassay variation = 0.05, for miR-499-5p slope = -3.37, y-intercept = 39.43, r^2^ = 0.97, efficiency = 98%, linear range = 2.6*10^6^-160 copies, LOD =160 copies, Ct standard deviation at LOD = 0.11, intraassay variation = 0.04.

When preparing RNA from plasma, the yields are insufficient for proper quantitation with e.g. NanoDrop [21]. Therefore, equal volumes of RNA preparation, rather than equal RNA amounts, was used as input in the cDNA synthesis. Normalization for variations in RNA input was conducted using miR-17, which is stable and abundant in plasma and is unaffected by myocardial damage [21]. In our samples, miR-17 varied little between patients, with a coefficient of variation of 5.7%. To confirm that miR-17 was stable across patient groups a one-way ANOVA was conducted on raw Ct values and the means did not differ significantly between groups (p = 0.698). Samples where miR-17 was not detected were regarded as poor quality RNA and those patients were excluded from the analysis (n = 10).

The threshold cycle (C_t_) was defined manually for each plate and was set where all assays were in the log linear phase and the threshold was above background for all assays. C_t_-values > 37 were considered as unspecific amplification. All samples where miR-1, miR-208b or miR-499-5p was not detected were given the C_t_-value 37 (1% of patients for miR-1, 33% for miR-208b and 24% for miR-499-5p). To adjust for differences in the amount of total RNA in each sample, C_t_-values were normalized against miR-17 (∆Ct) and transformed into quantities using the formula 2^-∆Ct^. An inter-plate calibrator sample was used to normalize for run-to-run variation.

### Statistical analysis

Plasma miRNA quantity was transformed using the natural logarithm as the distribution was positively skewed upon visual inspection. Patients whose blood samples had been taken >1 week after admission (n = 5) and patients whose admission date had not been registered (n = 2) were excluded from the analysis.

First, the distribution of each miRNA was compared across patient groups (STEMI, NSTEMI, non-MI) using one-way analysis of variance (ANOVA). Second, we determined the ability of miRNA level to discriminate between MI and non-MI patients, using Receiver Operator Characteristic (ROC) analysis. Third, we examined the long-term prognostic impact of elevated miRNA levels. The primary endpoint was predefined as death within 30 days of hospitalization (n = 5) or development of heart failure (clinical diagnosis (n = 25), an ejection fraction < 40% (n = 65) or development of cardiogenic shock (n = 2)) during follow-up. ROC analysis was then used to assess and compare the prognostic value of miRNAs and Troponin T using the primary endpoint as state variable. Finally, correlation of miRNA levels and LVEF and Troponin T (TnT) was tested using Spearman’s rank correlation coefficient. All statistical analyses were performed in SPSS Statistics v.19.0.0 (IBM, Armonk, USA).

### Ethics

All patients gave their informed consent before being included in the study. The study was approved by the local ethics committee of Skane University Hospital and carried out in compliance with the Declaration of Helsinki.

## Results

### Circulating miR-208b and miR-499-5p specifically reflect myocardial damage

**Figure 1:**
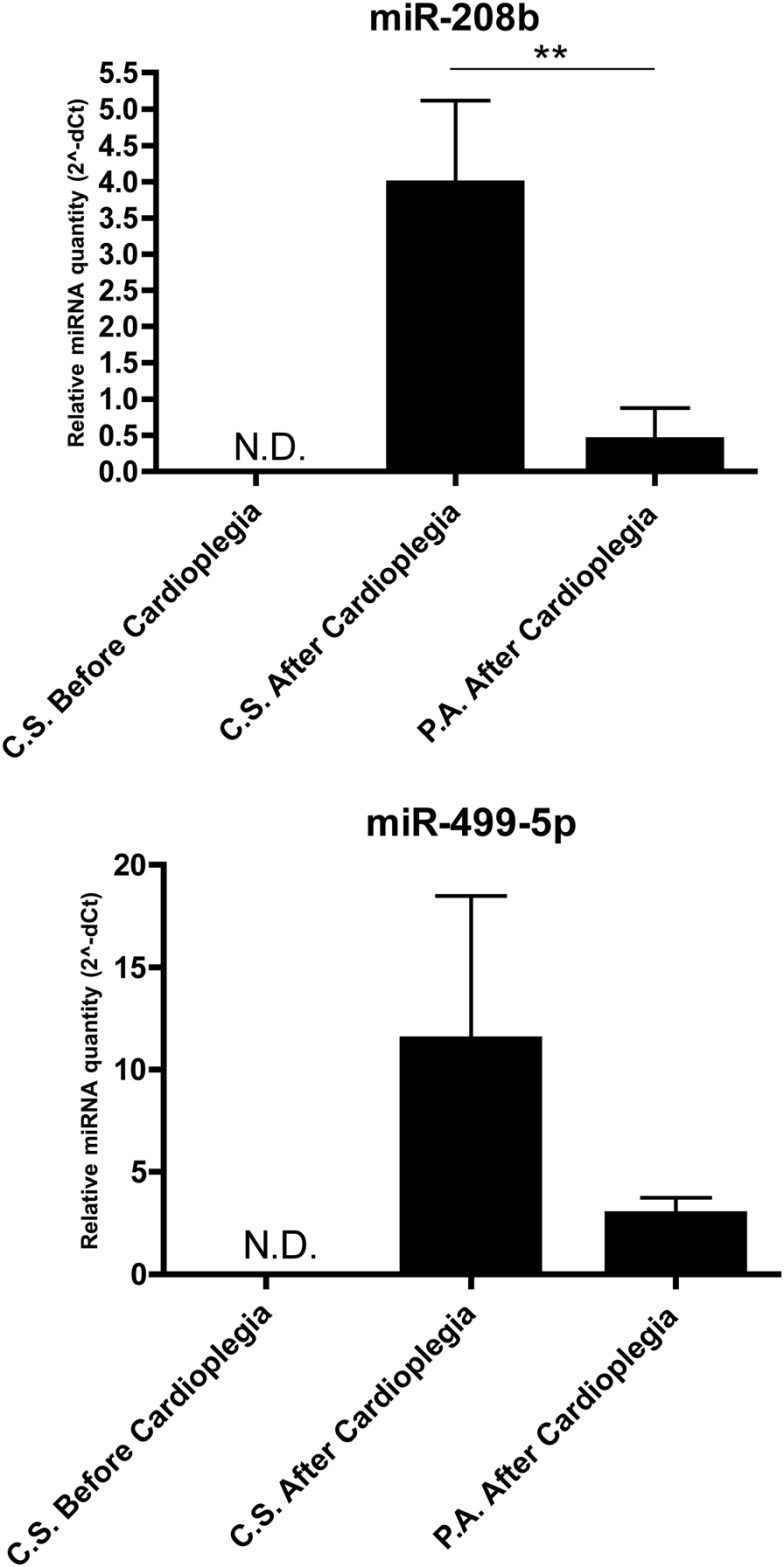
**Circulating miRNA levels in CABG patients.** MiRNA levels relative to miR-17, transformed into quantities using the formula 2^-dCt^. Error bars represent standard error of the mean (SEM). N.D. = not detectable, C.S. = coronary sinus, P.A. = peripheral artery. **p < 0.01.

To confirm that miR-208b and miR-499-5p are released directly from the myocardium as a result of tissue damage, we analysed miRNA levels in the coronary sinus of CABG-patients before and immediately after cardioplegia (see Figure 1). miR-208b and miR-499-5p were undetectable in the coronary sinus before cardioplegia but became readily detectable immediately after. The levels of miR-208b were significantly higher in the coronary sinus than in the periphery after cardioplegia (p < 0.01). For miR-499-5p, levels were higher in the coronary sinus than in the periphery but the difference did not reach statistical significance.

### Levels of circulating miRNA in ACS patients

**Figure 2:**
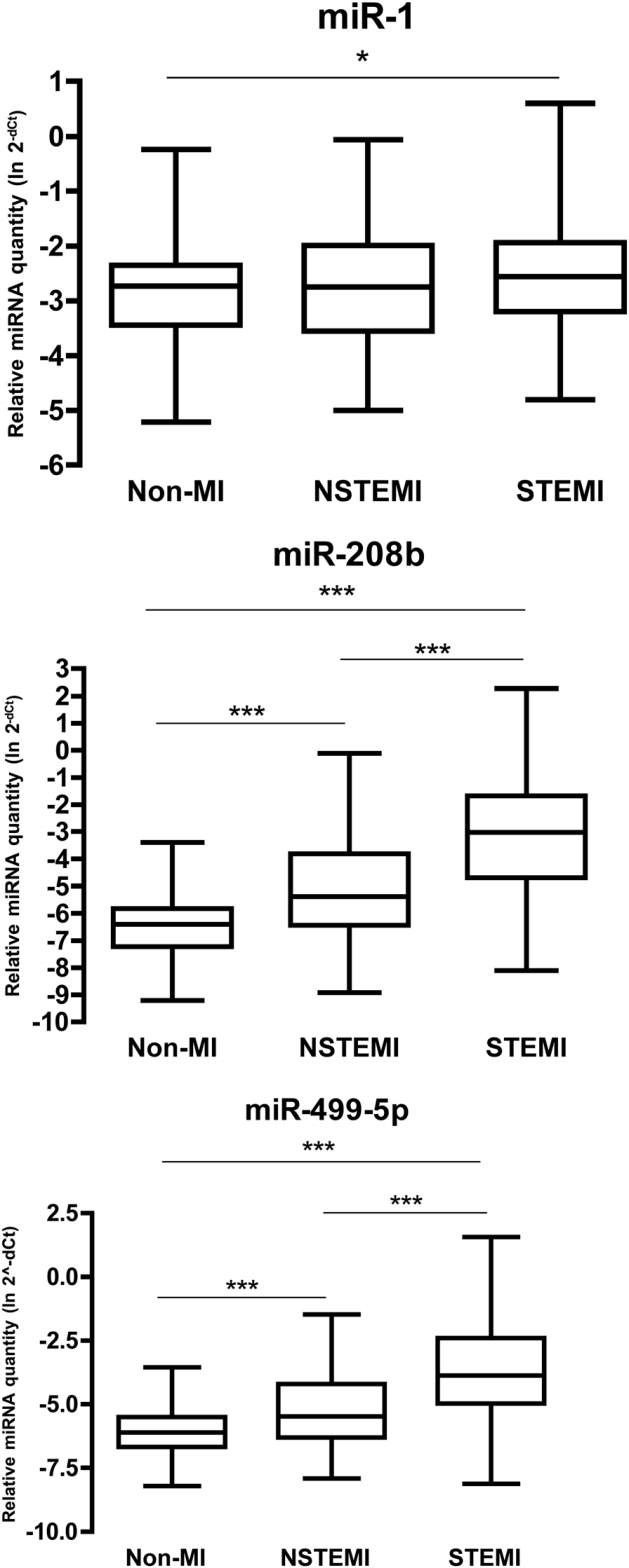
**MiRNA levels across patient groups.** MiRNA levels relative to miR-17, transformed into linear form using the formula 2^-dCt^. All quantities have been transformed using the natural logarithm. The box extends from the 25th percentile to the 75th percentile with a line at the median. The whiskers indicate the highest and the lowest value in each group. *p < 0.05, ***p < 0.001.

A total of 424 patients with suspected ACS were included and underwent measurement of plasma levels of miR-1, -208b, and -499-5p. Of these, 17 samples were excluded due to poor RNA quality (n = 10), excessive time from admission to sampling (>1 week, n = 5) or failure to register admission date (n = 2). A majority (76.2%) of patients were men and the median age was 65 years. 173 patients were diagnosed with STEMI, 146 with NSTEMI and 88 as non-MI. In the non-MI group, 64% were diagnosed with unstable angina, 22% with stable angina and 14% were defined as general chest pain. Patient characteristics are summarized in Table 2. Distributions of each miRNA across patient groups are shown in Figure 2. The levels of miR-208b and miR-499-5p were significantly higher in both NSTEMI and STEMI patients compared to non-MI patients (p < 0.001). miR-208b and -499-5p were also significantly higher in STEMI than in NSTEMI patients (p < 0.001). The levels of miR-1 were slightly higher in NSTEMI patients compared to the non-MI group (p < 0.05).

### Diagnosis of myocardial infarction

**Figure 3:**
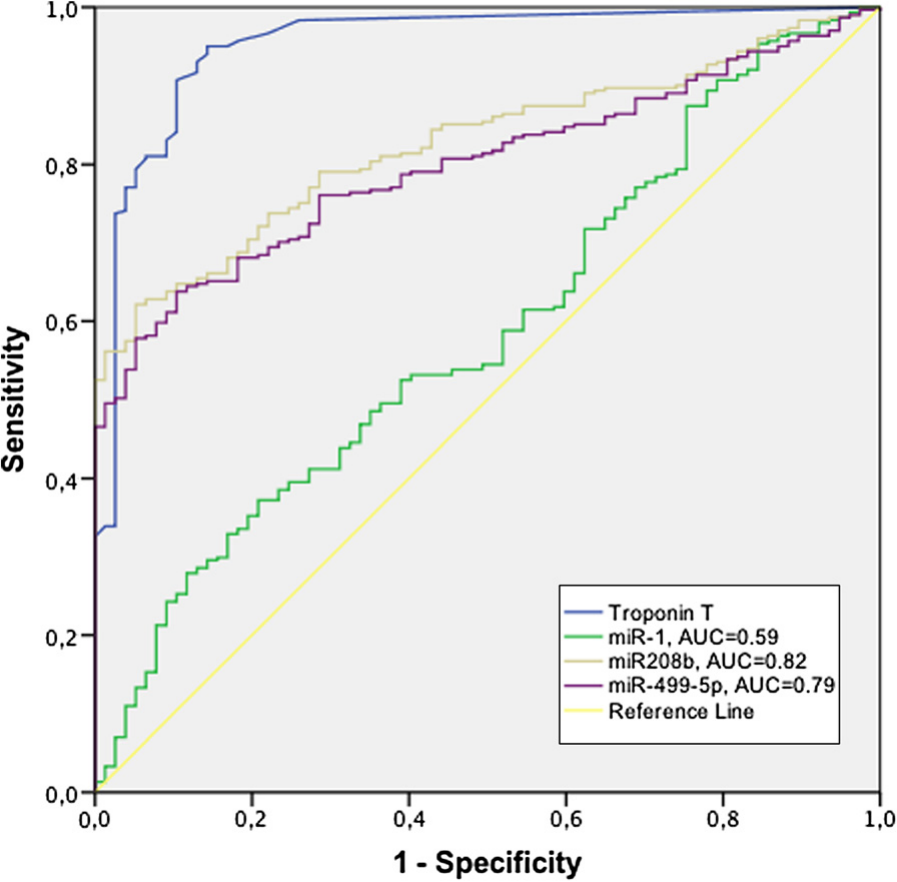
**Discrimination of myocardial infarction.** ROC curves describing the discrimination of patients diagnosed with MI from non-MI patients with circulating miRNA levels as well as Troponin T.

ROC curves describing the discrimination of patients diagnosed with MI (n = 319) from non-MI patients (n = 88) with circulating miRNA levels as well as the levels of the current cardiac marker Troponin T are shown in Figure 3.

The best discriminatory characteristics for a miRNA were observed for miR-208b, with an area under the ROC curve (AUC) of 0.82. miR-499-5p had an AUC of 0.79 whereas the AUC for miR-1 was 0.57. The current cardiac marker Troponin T (TnT) had an AUC of 0.95.

### Prognosis after myocardial infarction

**Figure 4:**
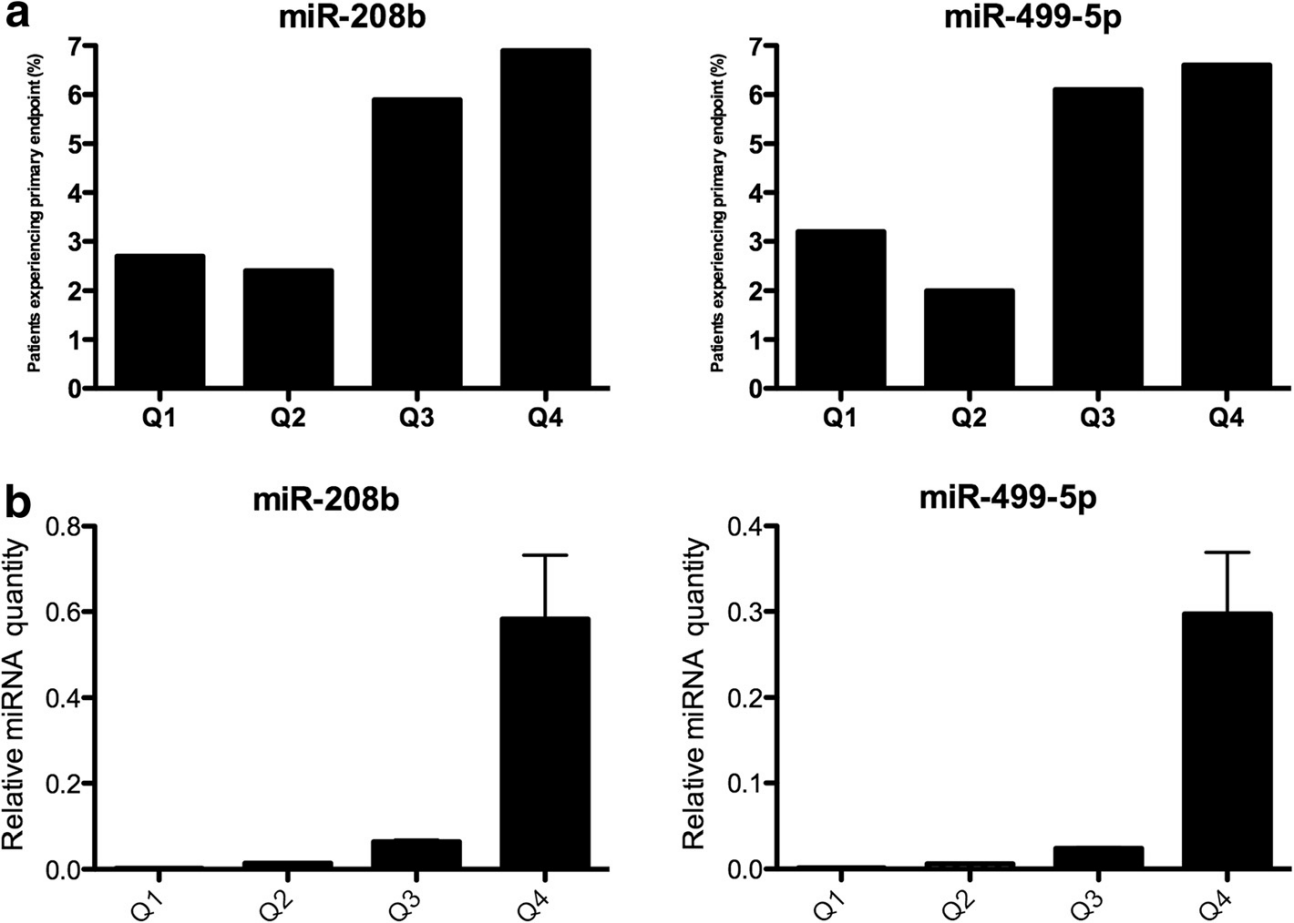
**Association of miRNA with the primary endpoint. a**) The level of each miRNA was divided into quartiles. Each bar represents the proportion of patients (%) in each quartile who experienced the primary endpoint. **b**) Levels of miRNA in each quartile, expressed relative to miR-17 and transformed into linear form using the formula 2^-dCt^.

A total of 74 patients (18%) experienced the primary endpoint. Plasma levels of miR-208b and miR-499-5p were associated with the primary endpoint. Odds ratios, adjusted for age, sex, time from admission to sampling and calculated per quartile, were 1.79 (95% CI = 1.38-2.33, p = 1 × 10^-5^) for miR-208b and 1.70 (95% CI = 1.31-2.20, p = 5 × 10^-5^) for miR-499-5p. When adjusting also for TnT, statistical significance was lost (p = 0.21 for miR-208b and 0.11 for miR-499-5p, respectively). Figure 4a depicts the proportion of patients in each quartile that experienced the primary endpoint. The relative miRNA levels in each quartile are shown in Figure 4b. In patients with MI (n = 319), miR-208b and miR-499-5p were still significantly associated with the primary endpoint (OR = 1.71, 95% CI = 1.25-2.34, p = 0.001 for miR-208b and OR = 1.58, 95% CI = 1.17-2.13, p = 0.003 for miR-499-5p, respectively) while there were no significant associations in the non-MI group (OR = 1.55, 95% CI = 0.55-4.40, p = 0.41 for miR-208b and OR = 1.64, 95% CI = 0.60-4.51, p = 0.33 for miR-499-5p, n = 88). In patients aged 75 or older (n = 89), the risk of experiencing the primary endpoint was higher than in younger patients. For miR-208b, OR = 2.20 (95% CI 1.26-3.82, p = 0.005) in patients >75 years old compared to 1.66 (95% CI 1.21-2.26, p = 0.001) for patients <75 years old. For miR-499-5p, the respective odds ratios were 1.99 (95% CI 1.20-3.32, p = 0.008) and 1.59 (95% CI = 1.17-2.16, p = 0.003), respectively. miR-1 levels were not significantly associated with the primary endpoint in any of the analyses.

**Figure 5:**
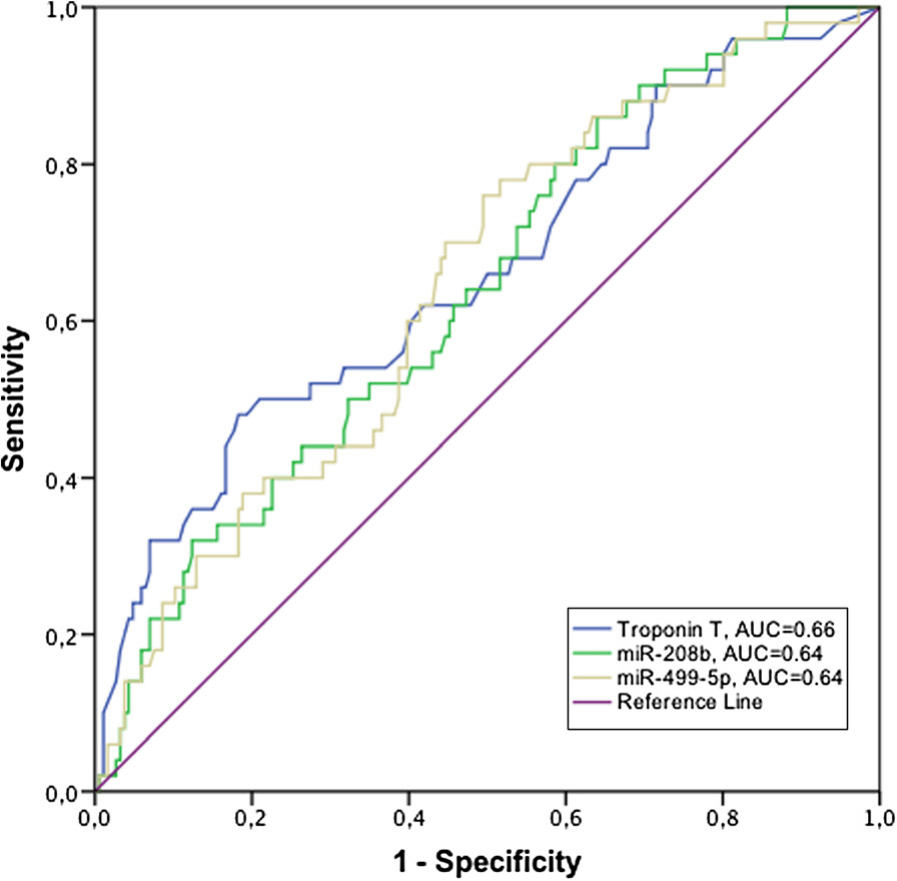
**Prognostic accuracy of miRNAs and Troponin T.** ROC curves for miR-208b, miR-499-5p and Troponin T with the primary endpoint as state variable.

To further assess the prognostic value of miR-208b and miR-499-5p compared to TnT, ROC analysis was performed with the primary endpoint as the state variable (Figure 5). Both miRNAs had similar accuracy as TnT (AUC = 0.64 for both miRNAs and 0.66 for TnT).

### Relation to conventional prognostic markers

In order to further evaluate the usefulness of circulating miRNA as cardiac biomarkers, we tested whether the levels of miR-1, -208b and -499-5p correlated with TnT. miR-208b and -499-5p were strongly correlated with TnT (r = 0.65, p < 1*10^-7^ and r = 0.62, p < 1*10^-7^) as well as with one another (r = 0.93, p < 0.0000001). Adjusting for age, sex and time from admission to sampling did not affect the level of statistical significance. miR-1 was weakly correlated with TnT (r = 0.12, p = 0.02), but lost statistical significance after adjustment for age, sex and sampling time. In subgroup analyses, TnT remained correlated with miRNA levels in MI patients (r = 0.57, p < 1*10^-7^ for miR-208b and r = 0.55, p < 1*10^-7^ for miR-499-5p) but not in non-MI patients (r = 0.1, p = 0.37 for miR-208b and r = 0.2, p = 0.08 for miR-499-5p).

We also investigated whether an increase in circulating cardio-enriched miRNA correlated with long-term myocardial systolic function, as measured by a LVEF. All miRNAs were weakly negatively correlated with LVEF (r = -0.11, p = 0.037 for miR-1; r = -0.16, p = 0.003 for miR-208b; r = -0.16, p = 0.003 for miR-499-5p). In subgroup analyses, LVEF was negatively correlated with miRNA in MI patients but not in non-MI patients. In multivariate analysis, the level of statistical significance for all correlations were unaffected by adjustment for age, sex and sampling time. Scatter plots for all correlations are shown in Additional file 1: Figure S1 and S2.

## Discussion

The notion of circulating miRNA as biomarkers for various diseases has been explored to a considerable extent in recent years. Tissue specificity, rapid release dynamics and stability in blood [21-24] and urine [23] make miRNAs promising candidates for diagnostic and prognostic utility in a wide range of disease states. miRNA species expressed at particularly high levels in cardiac or skeletal muscle, such as miR-1, miR-133, miR-208a/b and miR-499-5p have been thought of as candidate biomarkers in ACS. Thus far, most studies related to these questions have been small and focused on the acute phase of the disease. We wanted to investigate whether circulating cardio-enriched miRNA are valuable both for diagnosis and prognosis in a larger cohort of patients with suspected ACS.

Although many studies have claimed that the presence of cardio-enriched miRNA in the circulation reflect myocardial damage[15,16,21,22], only one very recent study have tried to show direct evidence that these miRNA species are in fact released directly from human myocardium [25]. To address this, we sampled blood from the coronary sinus before and after cardioplegia and reperfusion in patients undergoing CABG. The presence of miR-208b and miR-499-5p in the coronary sinus immediately after, but not before cardioplegia, supports the idea that these miRNAs are in fact released directly from the myocardium following tissue damage. Although the conditions during CABG do not directly reflect the situation in MI, these are both states of circulatory standstill within the myocardium and a reperfusion injury that amounts to tissue stress and cell death. Our data provides indirect evidence that miR-208b and miR-499-5p are released specifically from the heart in man.

The levels of all three cardio-enriched miRNAs displayed the same pattern, with increased amounts in NSTEMI patients compared to non-MI and increased amounts in STEMI patients compared to NSTEMI. This partly contradicts the results of a recent study [26], where the level of miR-499 was not increased in STEMI/NSTEMI compared to non-MI. However, this is not entirely unexpected since the release pattern of miR-499-5p is slower than that of miR-1/-208b [23] and the samples were taken at a later time point in this study compared to the study by Widera et al. It is possible that the peak of miR-499-5p had not yet been reached at the time of sampling in that study.

Although MI patients could be discriminated from non-MI patients based on the plasma levels of miR-208b and miR-499-5p, the accuracy was well below that of the current gold standard cardiac marker, Troponin T. However, it is likely that the accuracy could be improved with faster blood sampling, considering the rapid dynamics of circulating cardio-enriched miRNAs [21] and the fact that 29% of the blood samples were taken more than 24 hours after admission. In fact, a subgroup analysis with only the samples that were taken within 24 hours showed a markedly improved diagnostic accuracy. AUC for miR-208b increased from 0.82 to 0.89 and from 0.79 to 0.87 for miR-499-5p. Another factor that hampers a direct comparison is that blood sampling for miRNA and TnT analysis was not consistently obtained at the same time point.

To evaluate whether circulating miRNA holds any prognostic value we evaluated a primary endpoint including death within thirty days of admission and development of heart failure. Plasma levels of miR-208b and miR-499-5p were associated with an increased risk of death or heart failure in MI patients (STEMI and NSTEMI) but not in non-MI patients and may thus provide some prognostic information following MI. An increased level of either of the three miRNAs also correlated with decreased systolic function, confirming the findings of our recent, smaller study [23]. It is likely that the release of cardio-enriched miRNAs into the bloodstream following myocardial damage is merely a by-product of necrotic cardiomyocytes. However, the possibility of a paracrine effect of these miRNA species, as e.g. shown for miR-126 in the context of atherosclerosis [13], which might exacerbate the conditions within the myocardium post-MI and increase the risk of death or development of heart failure, cannot be excluded.

During preparation of this manuscript one other large study evaluating the prognostic impact of cardio-enriched miRNAs on survival in the context of ACS was published [26]. miR-133 and -208b was associated with an increased risk of death within six months. As with the results presented herein, the association did not remain statistically significant after adjusting for another cardiac biomarker. These findings suggest that miRNA levels do not offer incremental prognostic information regarding risk of death or development of heart failure than the current cardiac markers. However, the most important potential utility of these new biomarkers lie in the rapid release dynamics and substantial rise in plasma levels. Additional studies are warranted to assess the utility of miRNAs in rapid diagnosis of MI in the emergency department and in the diagnosis of early reinfarction, where conventional biomarkers perform poorly. Additional studies are also warranted in patient groups where traditional cardiac markers are less reliable, for example in the elderly, and in patients with severe heart failure or renal failure and dialysis.

Our study also has limitations which merit consideration. First, the CABG patient cohort consists of only four individuals and the results regarding the origin of cardio-enriched miRNAs must be taken with caution. Second, as patients were recruited at the coronary care unit, our study did not include many patients presenting with possible ACS of lower likelihood to represent ischemic heart disease, which are more often treated in a general acute admissions unit. This may have attenuated the diagnostic performance of biomarkers, as patients transferred to the coronary care unit more often have concurrent cardiac disease or other complicating factors, which may be associated with increased biomarker levels. Third, comparisons of miRNA and Troponin T are complicated by the fact that these samples were not consistently obtained at the same time point.

## Conclusion

In conclusion, our findings indicate that cardio-enriched miRNAs are released from the myocardium in response to ischemia, that the levels of cardio-enriched miRNAs are increased in MI and establish association of increased miRNA levels with reduced systolic function after MI and risk of death or heart failure.

## Competing interest

The authors declare that they have no competing interests.

## Authors' contributions

OG - designed the study, carried out experiments, analyzed and interpreted data, wrote the manuscript. GS - analyzed and interpreted data, revised the manuscript for important intellectual content. KM - carried out experiments, revised the manuscript for important intellectual content. PA - analyzed and interpreted data, revised the manuscript for important intellectual content. AS - provided clinical samples, acquired data, revised the manuscript for important intellectual content. SB - provided clinical samples, acquired data, revised the manuscript for important intellectual content. DE - designed the study, analyzed and interpreted data, revised the manuscript for important intellectual content. All authors read and approved the final manuscript.

## Pre-publication history

The pre-publication history for this paper can be accessed here:

http://www.biomedcentral.com/1471-2261/13/12/prepub

## Supplementary Material

Additional file 1 Gidlof et al, “Circulating cardio-enriched miRNAs are associated with long term prognosis following myocardial infarction.” Click here for file
