# Supplementary material for: Circulating cardio-enriched microRNAs are associated with long-term prognosis following myocardial infarction
Source: BMC Cardiovasc Disord. 2013 Feb 28;13:12. doi: 10.1186/1471-2261-13-12 (PMC3598930; doi:10.1186/1471-2261-13-12)
Supplement: Additional file 1 — Gidlof et al, “Circulating cardio-enriched miRNAs are associated with long term prognosis following myocardial infarction.” [file 1471-2261-13-12-S1.pdf]

Supplemental Data, Gidlof et al, "Circulating cardio-enriched miRNAs are associated with long term prognosis following myocardial infarction"

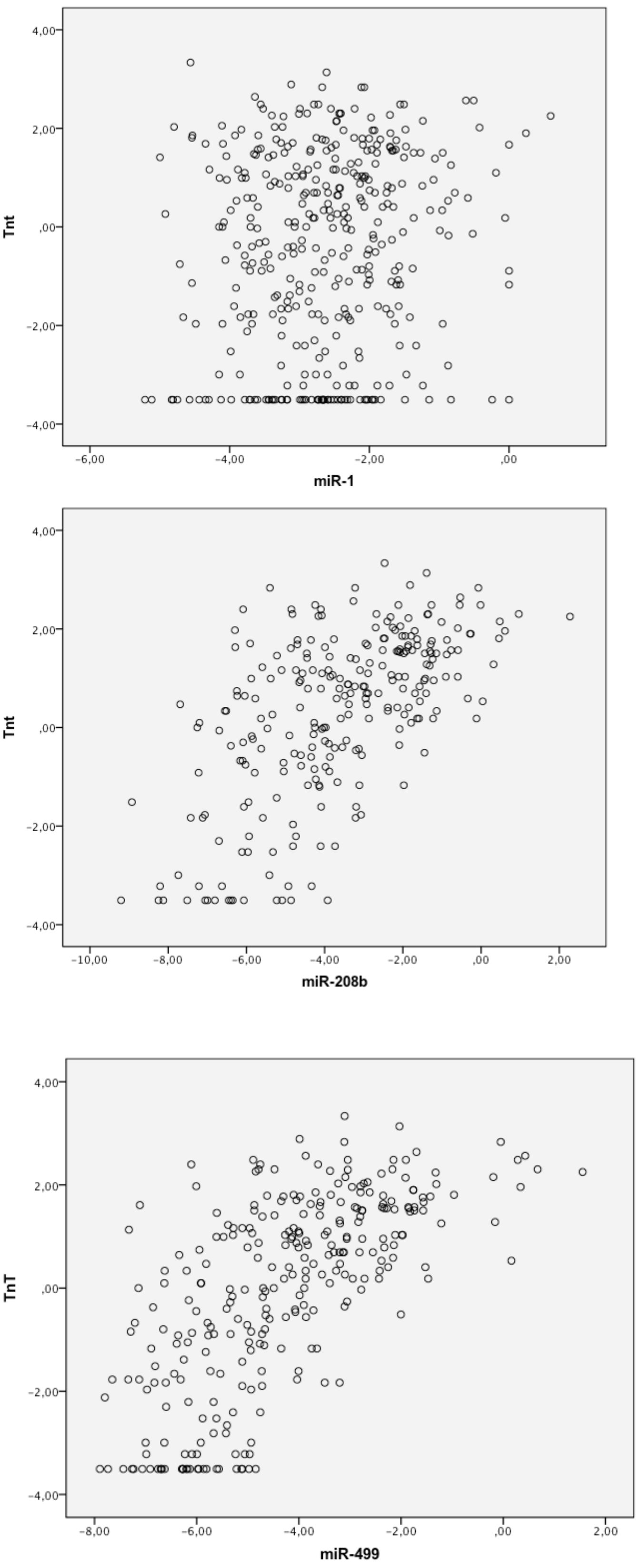

Supplemental Figure 1. Scatter plots for miRNAs and Troponin T.

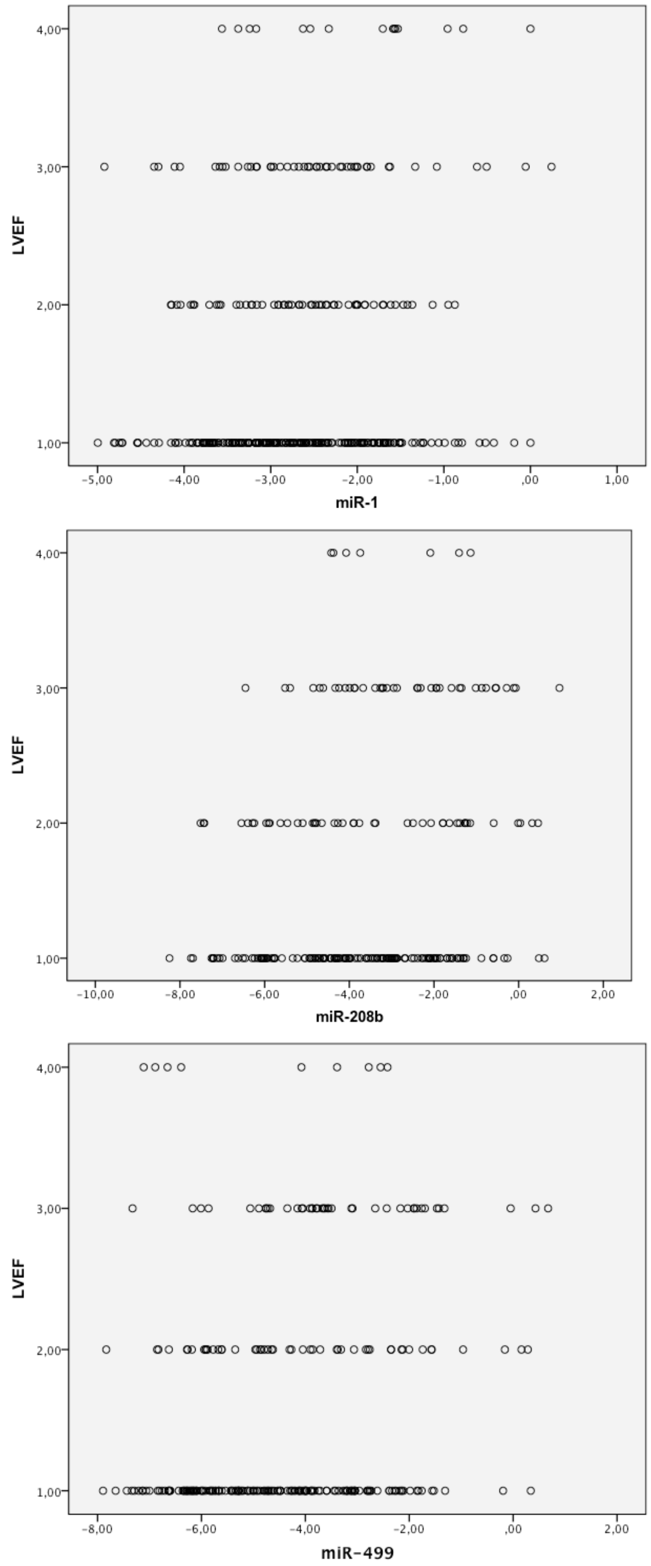

Supplemental Figure 2. Scatter plots for miRNAs and LVEF.
